# Biomineralization of cyanobacteria *Synechocystis pevalekii* improves the durability properties of cement mortar

**DOI:** 10.1186/s13568-022-01403-z

**Published:** 2022-05-19

**Authors:** Navneet Sidhu, Shweta Goyal, M. Sudhakara Reddy

**Affiliations:** 1 Department of Biotechnology, Thapar Institute of Engineering & Technology, Patiala, 147004 Punjab India; 2 Department of Civil Engineering, Thapar Institute of Engineering & Technology, Patiala, 147004 Punjab India

**Keywords:** Cyanobacteria, *Synechocystis pevalekii*, Calcium carbonate precipitation, Sand consolidation, Compressive strength, Water absorption, Mortar

## Abstract

Microbially induced calcium carbonate precipitation (MICCP) is considered a novel eco-friendly technique to enhance the structural properties of cementitious-based material. Maximum studies have emphasized using ureolytic bacteria to improve the durability properties of building structures. In this study, the role of photoautotrophic bacteria *Synechocystis pevalekii* BDHKU 35101 has been investigated for calcium carbonate precipitation in sand consolidation, and enhancing mechanical and permeability properties of cement mortar. Both live and UV-treated *S. pevalekii* cells were used to treat the mortar specimens, and the results were compared with the control. The compressive strength of mortar specimens was significantly enhanced by 25.54% and 15.84% with live and UV-treated *S. pevalekii* cells at 28-day of curing. Water absorption levels were significantly reduced in bacterial-treated mortar specimens compared to control at 7 and 28-day curing. Calcium carbonate precipitation was higher in live-treated cells than in UV-treated *S. pevalekii* cells. Calcium carbonate precipitation by *S. pevalekii* cells was confirmed with SEM-EDS, XRD, and TGA analysis. These results suggest that *S. pevalekii* can serve as a low-cost and environment friendly MICCP technology to improve the durability properties of cementitious materials.

## Keypoints

- Cyanobacterial *Synechocystis pevalekii* cells induced calcification on cement mortar.
- Both live and UV-treated cells increased the compressive strength and reduced the water absorption.
- Biomineralization by *S. pevalekii* serves as a low-cost and eco-friendly MICCP technology.

## Introduction

Microbially induced calcium carbonate precipitation (MICCP) is a biochemical process caused by microorganisms, resulting in calcium carbonate as a product. For the last two decades, this process has drawn significant attention from scientific researchers in the area of environment, biotechnology, and civil engineering because of its eco-efficiency over the expensive chemical treatments for concrete repair and restoration (Dhami et al. 2013). The most studied technique in these processes is ureolysis by heterotrophic bacteria due to its high calcium carbonate precipitating ability (Dhami et al. 2013). Ureolytic bacteria can induce calcium carbonate by accumulating a urease enzyme that utilizes urea as a substrate and calcium as a source for mineralization. However, one of the significant setbacks in ureolysis is the release of ammonia gas, a harmful air pollutant with a pungent smell. The ammonia formation results in nitrogen oxide releasing into the atmosphere, producing a foul odor (De Muynck et al. 2010). Moreover, excess ammonium in the concrete increases the risk of salt damage by converting it into nitric acid (Seifan et al. 2016). Scientists are hence, continuously looking for another alternative to overcome this drawback. The use of phototrophic bacteria can serve as a potential source alternative to heterotrophic bacteria in enhancing the durability properties of building structures.

Recently, an eco-efficient approach involving photoautotrophic bacteria has gained attention in utilizing them in improving the durability properties of building materials (Zhu et al. 2017, 2018). Unlike ureolytic bacteria, photoautotrophic bacteria use carbon dioxide and sunlight as energy instead of urea and produce carbonate crystals. Photoautotrophic bacteria such as cyanobacteria can induce carbonate precipitation in aquatic and terrestrial environments (Douglas and Beveridge 2006; Dittrich and Sibler 2010; Kranz et al. 2010). Therefore, the utilization of cyanobacteria for the biomineralization process in cementitious structures has attracted many researchers (Zhu et al. 2017, 2018).

Phylogenetically cyanobacteria are diverse and found in moist soil, marine or freshwater, extreme habitats like hot springs, hypersaline water, and deserts. The most distinctive characteristic of cyanobacteria is its ability to survive in a minimum requirement of light, carbon dioxide (CO_2_), and water (Singh et al. 2016). Due to the exchange process of HCO_3_¯ /OH¯ ions across the cell membrane during CO_2_ fixation, the pH increases, creating an alkaline microenvironment that favors the carbonate precipitation (Han et al. 2013). The photosynthetic reaction of cyanobacteria is catalyzed by RUBISCO-carboxylase and carbonic anhydrase enzymes. The CO_2_ enters the cyanobacterial cell through the HCO_3_¯/Na^+^ symporter, which is further converted to HCO_3_¯. In the carbon concentration mechanism (CCM.), HCO_3_¯ in the cytosol is carried to the carboxysome and converted into CO_2_ through carbonic anhydrase activity (Eq. 1). As a result, the OH¯ is released across the cell membrane, increasing the pH of the cell exterior (Kamennaya et al. 2012).

1 $${\text{CO}}_{{\text{2}}} + {\text{ H}}_{{\text{2}}} {\text{O }} \leftrightarrow {\text{ H}}^{ + } + {\text{ HCO}}_{3} {^{ - }}$$

Calcium carbonate precipitation is an extracellular process that occurs either in exopolysaccharide sheath (EPS) or in the proteinaceous surface layer (S-layer), which are surrounding the cells (Han et al. 2013). Consumption of H^+^ ions due to carbonic anhydrase activity increases alkalinization at EPS or S-layer. The EPS layer acts as a nucleation site for calcium carbonate binding. The calcium carbonate precipitation proceeds by following reactions.

2 $${\text{Ca}}^{{{\text{2}} + }} + {\text{2HCO}}_{3} {^{ - }} \leftrightarrow {\text{CaCO}}_{{\text{3}}} + {\text{ CO}}_{{\text{2}}} + {\text{ H}}_{{\text{2}}} {\text{O}}$$

3 $${\text{Ca}}^{{{\text{2}} + }} + {\text{CO}}_{{\text{3}}} ^{{2 - }} \leftrightarrow {\text{CaCO}}_{{\text{3}}}$$

Precipitation of calcium carbonate by cyanobacteria such as *Oscillatoria* sp., *Synechococcus* sp., and *Synechocystis* sp., has been reported under laboratory conditions (Gnanasekaran 2014; Han et al. 2017; Paulo et al. 2018). Recently, the carbonate precipitation on cementitious structures by cyanobacteria has also been reported. Researchers reported the carbonate precipitation on the surface of cement mortar by cyanobacterial species such as *Gloeocapsa* sp. and *Synechococcus* sp. (Zhu et al. 2017, 2018; Srinivas et al. 2021) investigated the crack healing of cement mortar by using two microalgal species, *Synechococcus elongatus* and *Spirulina platensis*. Their results suggest that both the species can heal the cracks and improve the durability properties of the cement mortar with maximum potential by *S. platensis*. Though these studies show the potential use of phototrophic bacteria for crack healing and improvement of durability properties, the application of algae is still in the process of development, and more research needs to be done.

The present study was aimed to investigate the biomineralization potential of cyanobacteria, *Synechocystis pevalekii* isolated from the marine ecosystem. The biocalcification potential of *S. pevalekii* was studied by conducting experiments in laboratory conditions. The strength and permeability properties of cement mortar were investigated by treating them with live- and UV killed cells of *S. pevalekii*. The morphology of calcium carbonate crystals formed was determined using by scanning electron microscope (S.E.M.) and physio-chemical analysis by Energy-dispersive X-ray spectroscopy (EDX), X-Ray Diffraction (XRD), and Thermogravimetric analysis (TGA).

## Materials and methods

### Bacterial strain and cultivation conditions

The cyanobacterial strain, *Synechocystis pevalekii* BDHKU 35,101, isolated from the marine ecosystem (Bhuvaneshwari et al. 2017), was procured from the National Facility for Marine Cyanobacteria, Bharathidasan University Tiruchirappalli, Tamil Nadu, India. *Synechocystis pevalekii* cells are spherical, without individual mucilage envelope. The *S. pevalekii* cells were grown in BG 11 medium (Rippka et al. 1979), which has the following ingredients (g/l): NaNO_3_ 1.5 g, K_2_HPO_4_ 0.04 g, MgSO_4_·7H_2_O 0.075 g, CaCl_2_·2H_2_O 0.036 g, Citric acid 0.006 g, Ferric ammonium citrate 0.006 g, EDTA (disodium salt) 0.001 g, Na_2_CO_3_ 0.02 g, Trace metal mix A5 1.0 ml. (H_3_BO_3_ 2.86 g, MnCl_2_·4H_2_O 1.81 g, ZnSO_4_·7H_2_O 0.222 g, NaMoO_4_·2H_2_O 0.39 g, CuSO_4_·5H_2_O 0.079 g, Co(NO_3_)_2_·H_2_O 49.4 )

### Sand consolidation

**Fig. 1:**
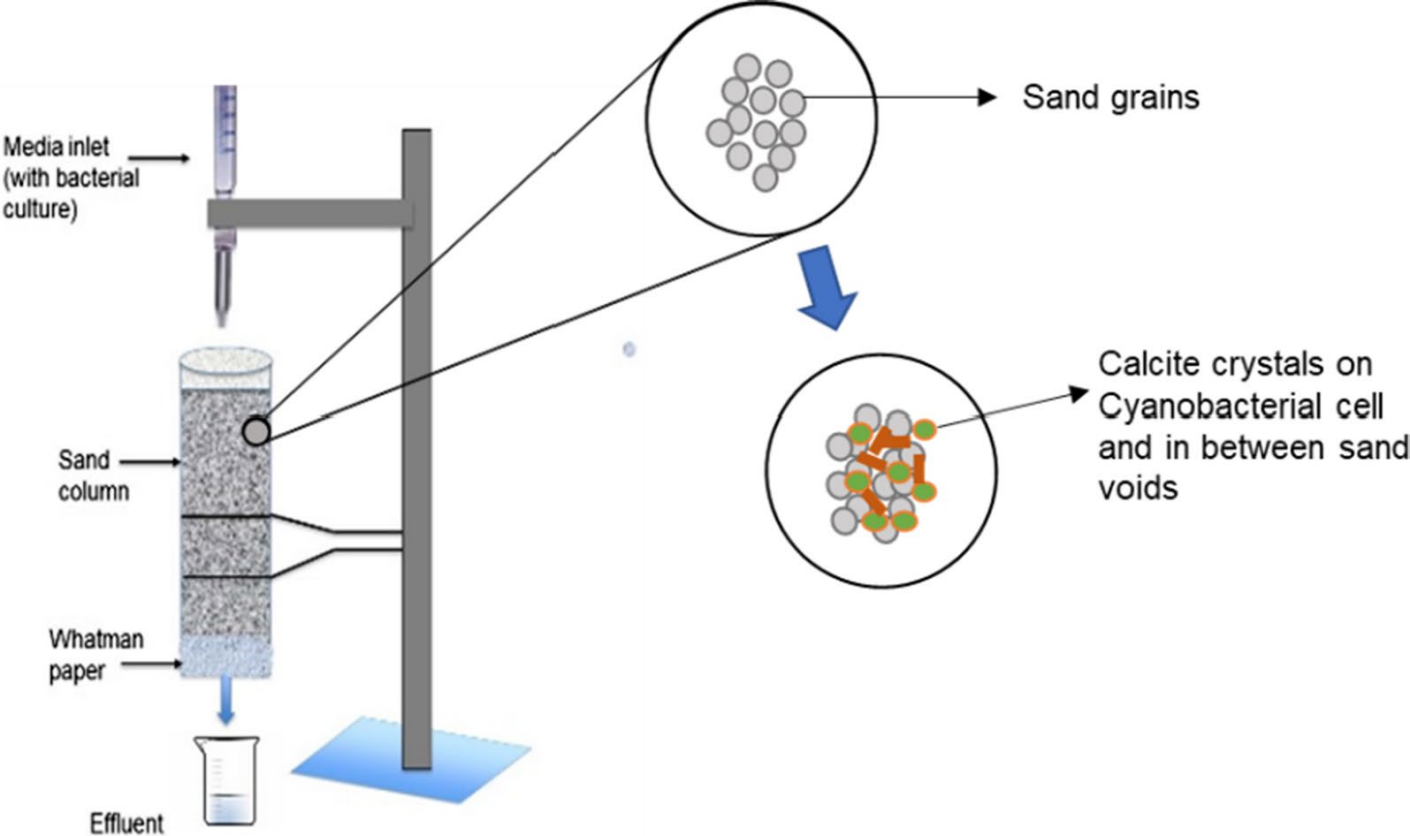
Schematic representation of sand column experiment

The sand consolidation experiment was performed using live (LT), and UV treated UVT) cells to determine the binding property of crystals. For UV treatment, *S. pevalekii* cell suspension was exposed to UV light for 1 h. For the sand column, locally available clean, dry, well-graded, natural river sand was used. The sand was sieved through 0.3 mm mesh and autoclaved to remove the indigenous microflora (Hamamura et al. 2006). The LT and UVT cells were taken and resuspended in 25 ml sterilized calcification media and prepared as shown in Table 1. For the preparation of sand columns, the slurry of sand keeping the moisture content of 25% was filled into a plastic tube (height 7.6 cm; diameter 3.8 cm) (Fig. 1). The bottom portion of each column was wrapped with Whatman filter paper. A burette was attached at the top of the plastic column to drop calcification media at a constant amount through the column. The control sets were made similar without the bacterial cells, while other specimens had the bacterial inoculum mixed. Outline for the experimental setup and calcifying solution used are presented in Table 1. All the columns were fed with 25 ml calcification media twice with a 12-h duration at room temperature for 14 days. The effluent was collected from the bottom of the sand columns in the beaker at regular intervals of 0, 2, 4, 6, 8, 10, 12, and 14 days to estimate the flow rate and pH to assess the activity of cyanobacterial cells within the sand columns. The sand column was opened on day-15, and the consolidated sand column was dried in an oven at 70 °C for 24 h. Samples were digested in 1.0 M hydrochloric acid at 40 °C under continuous stirring and were divided into three layers to estimate the amount of precipitation in each layer using the EDTA titration method (Stocks-Fischer et al. 1999). A small sample of upper consolidated sand layer was taken further for microstructural analysis (SEM-EDS).

**Table 1:** Sand consolidation experiment set-up with different calcifying media

Groups	Composition of sand column	Calcifying media
Live cells (LT)	Sand (0.3 mm) + bacterial cells + CaCl_2_ + NaHCO_3_	Bacterial cells + CaCl_2_ (100mM) + NaHCO_3_ (50 mM)
UV treated cells (UVT)	Sand (0.3 mm) + bacterial cells + CaCl_2_ + NaHCO_3_	Bacterial cells + CaCl_2_ (100mM) + NaHCO_3_ (50 mM)
Control	Sand (0.3 mm) + deionized water	Deionized water

### Experiments with mortar specimens

Mortar cubes of 70.6 × 70.6 × 70.6 mm were cast using an ordinary Portland cement (43 Grade) according to the IS: 8112 (2013). Locally available natural river sand was used as fine aggregate corroborative to Zone II (IS: 383 2016). Cement mortar mix was prepared by using cement:sand in the ratio of 1:3 (w/w) and water to cement ratio (w/c) of 0.47 as per IS: 4031 (2005). To cast bacterial-treated mortars, both live and UV-treated *S. pevalekii *(4 × 10^8^ cells/ml) culture was used instead of water. The bacterial culture to cement ratio was also maintained at 0.47. Freshly mixed ingredients at the plastic stage were immediately transferred into iron moulds in the casting room at room temperature of 27 ± 2 °C. After 24 h, the specimens were demolded and cured till the testing age. Different treatments, materials used, and the method of curing are mentioned in Table 2. The mortar specimens were divided into three groups. The first group was subjected to live *S. pevalekii * cells, the second group with UV treated culture, and the third group without bacterial treatment. All the mortar specimens were cured for 3-, 7-, and 28-day with their respective medium as mentioned in Table 2. Bacterial-treated specimens were initially submerged in live, and UV killed culture for 24 h and then in BG11 medium with 50 mM NaHCO_3_ and 100 mM CaCl_2_ in a plastic container at room temperature of 30 ± 2 °C for different curing periods. All specimens were exposed to a light intensity of 1200 lux for 12 h and kept under darkness for 12 h. For 28-day curing specimens, the cubes were incubated in culture for 24 h at the beginning of every week and then transferred to BG11 medium with 50 mM NaHCO_3_ and 100 mM CaCl_2_. At the end of the experiment, mortar specimens were dried at 72 °C for 24 h and analyzed for various parameters.

**Table 2:** Outline of different sets of mortar specimens and mechanism of curing treatment

Specimens	Material used	Mechanism of curing
Control	Cement:sand: 1:3; water/cement ratio 0.47	Water curing for 3,7 and 28 days
LT	Cement:sand: 1:3; live *S. pevalekii* culture/cement ratio 0.47	Curing for 3,7 and 28^a^ days in BG11 medium supplied with 100 mM CaCl_2,_ 50 mM NaHCO_3_.
UVT	Cement:sand: 1:3; UV-treated *S. pevalekii* culture/cement ratio 0.47	Curing for 3,7 and 28^a^ days in BG11 medium supplied with 100 mM CaCl_2,_ 50 mM NaHCO_3_

^a^Mortar cubes were incubated in culture for 24 h. at the beginning of every week and then transferred to medium

### Testing procedures

The compressive strength was measured with an automatic compression testing machine. A sorptivity test was performed based on the RILEM 25 P.E.M. (II-6) as described in Achal et al. (2011b). Thermogravimetric analysis (TGA) was performed to determine calcium carbonate precipitation in mortar specimens. Briefly, the sample was broken, crushed, and powdered into homogenous particle sizes, and 20 mg of mortar powder was taken in a TGA holder and increased the temperature constantly up to 850 °C with a heating rate of 10 °C/min. Nitrogen (10 ml/min) was used as a purge gas, and the loss of weight (%) of the sample during heating was plotted against temperature (°C). The weight loss in the temperature range of 550–750 °C corresponds to CaCO_3_ decomposition. Scanning electron microscopy (SEM) (ZEISS EVO 50) was performed for the samples at 28-day, and the elemental composition of microstructural crystals was identified with energy-dispersive X-ray spectroscopy (EDX.). Samples were finely polished and gold-coated with a sputter coating. X-ray diffraction (XRD) was performed using Bruker D8 X-ray diffractometer with a Cu anode (40 kV and 30 mA) and scanning from 20° to 60° at a 2θ angle.

### Statistical analysis

All the experiments were performed in triplicates. Data were analyzed by analysis of variance, and the significant differences among the means values were compared by Tukey’s test at P < 0.05. GraphPad Prism 5.1 software was used for analyzing the data.

## Results

### Sand consolidation

**Fig. 2:**
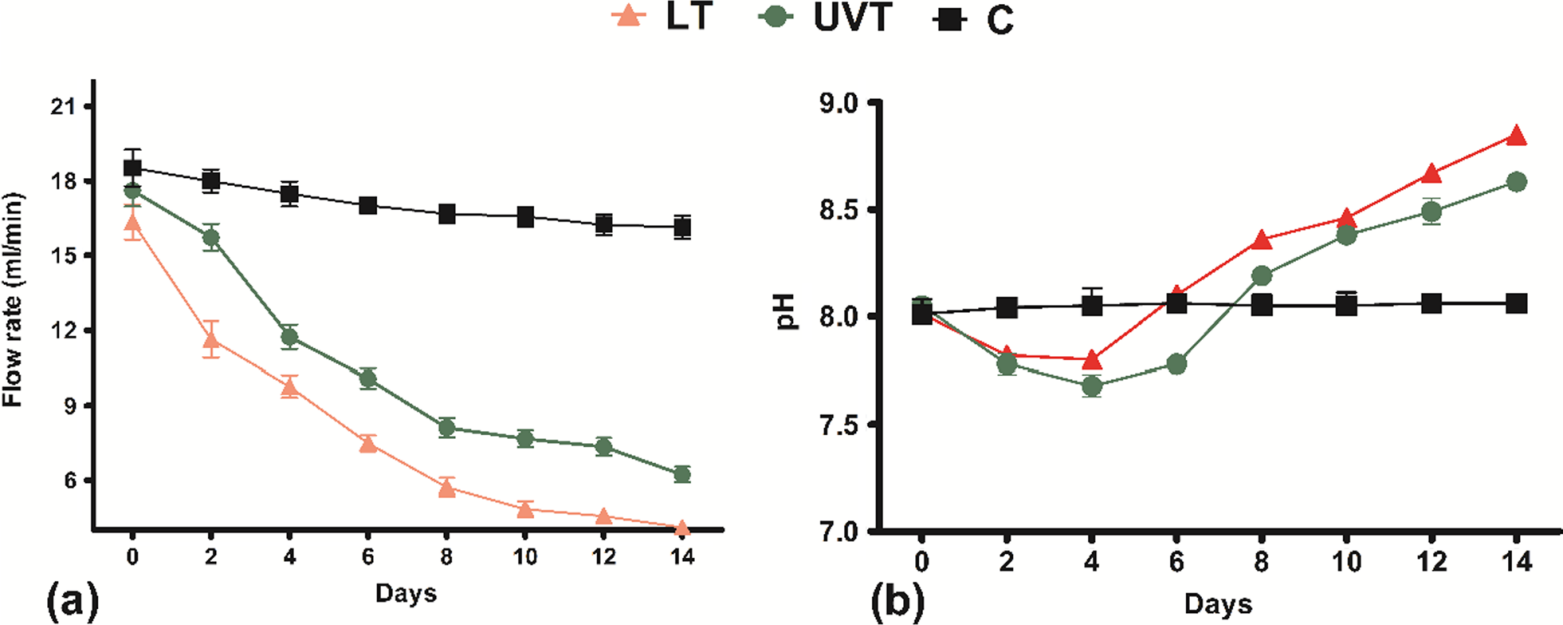
Estimation of **a** the flow rate of the calcifying solution **b** pH of the effluent solution injected through sand columns till 14 days

To study the binding property of the calcium carbonate precipitated by *S. pevalekii* cells, the sand consolidation experiment was performed with both LT and UVT cells of *S. pevalekii*. The sand column was continuously fed with BG11 nutrient media as mentioned in Table 1 for 14 days. The sand columns were opened, and a solid cylindrical consolidated sand column was observed in cyanobacterial injected columns. The flow rate of the calcifying medium was measured at regular intervals till 14 days of consolidation. The flow rates of *S*. *pevalekii* treated columns and the control columns were almost the same initially. There was a 12.9% reduction in the control column flow rate after 14 days, which shows a marginal change in the control column’s structures due to the flow. However, the flow rate declined significantly in the LT and UVT columns, indicating that MICCP is causing gradual constraint of the flow channels. The flow rate was almost obstructed at the end of the 12th day. Initially, on the 0-day, the flow rate was estimated to be 16.38, 17.44, and 18.52 ml/min in experiment set—LT, UVT and C (control). The flow rate on the 14th day in the experiment set LT and UVT was 4.10 and 6.23 ml/min, respectively. Bacterial columns showed a maximum 40.2% reduction in the flow rate on the 4th day of study, increasing to 74.90% on the 14th day (Fig. 2a).

The change in pH of the effluent collected at the bottom of the injected calcifying medium was monitored at 0, 2, 4, 6, 8, 10, 12, and 14 days of the experiment. The pH of the effluent was in the range of 7.3–7.5 for LT while 7.6–7.8 for UVT at the end of the 14 days (Fig. 2b). Conversely, the pH of the effluent from control columns remained the same throughout the experiment, i.e., pH 8.1.

**Fig. 3:**
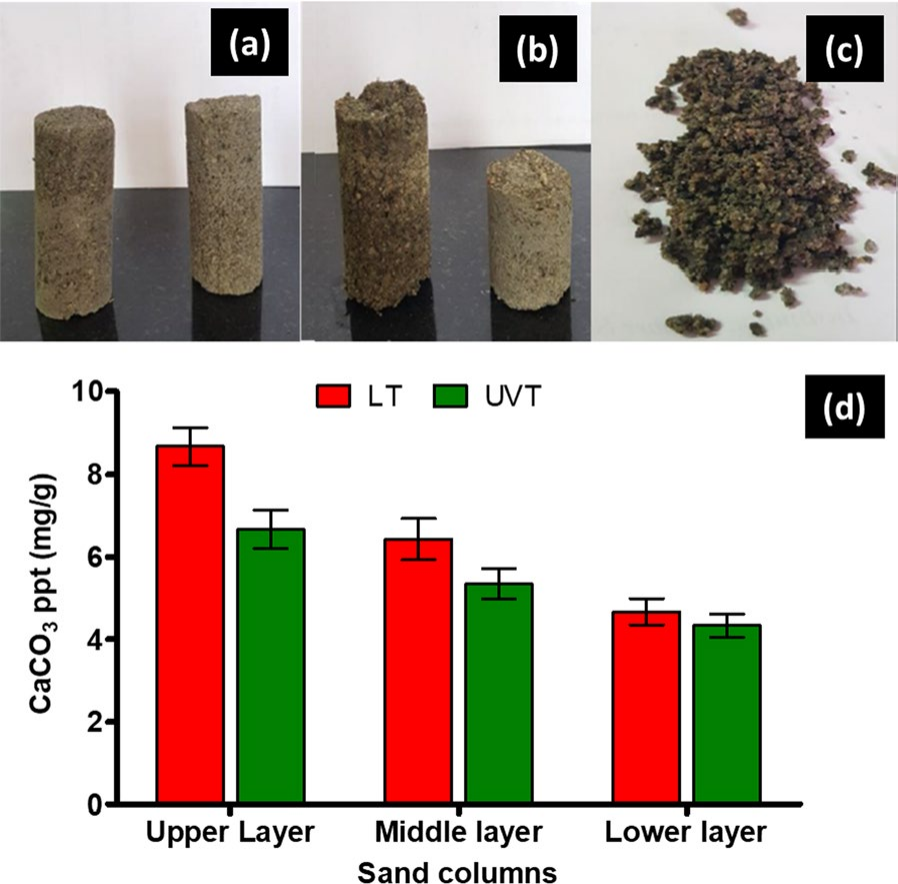
Sand columns consolidated by **a** live (LT) *S*. *pevalekii* cells, **b** UV treated (UVT) *S*. *pevalekii* cells, **c** control, and **d** calcium carbonate precipitation in three different layers of sand columns

The LT column resulted in complete consolidation of sand compared to control without bacteria (Fig. 3a). Sand columns treated with live cells were more compact and firm than UVT columns. Partial binding formed in UVT experimental setup, where about two-thirds of the sand was consolidated (Fig. 3b). There were clumps of sand on the top in UVT, whereas in control, the sand came out as it is without any consolidation (Fig. 3c). The consolidated sand column was further divided into three parts to estimate the calcium carbonate content. The upper part of the column in LT and UVT was dense and harder than the bottom parts. Figure 3d depicts the decreasing trend of calcium carbonate formation in the three layers of the sand column in LT and UVT columns. The maximum calcium carbonate content in the upper layer was 8.67 mg/g in LT treated column, and it was 6.67 mg/g in UVT column. The calcium carbonate precipitation in the middle part of sand columns was recorded as 6.43 mg/g, and 5.43 mg/g, respectively in LT and UVT columns.

**Fig. 4:**
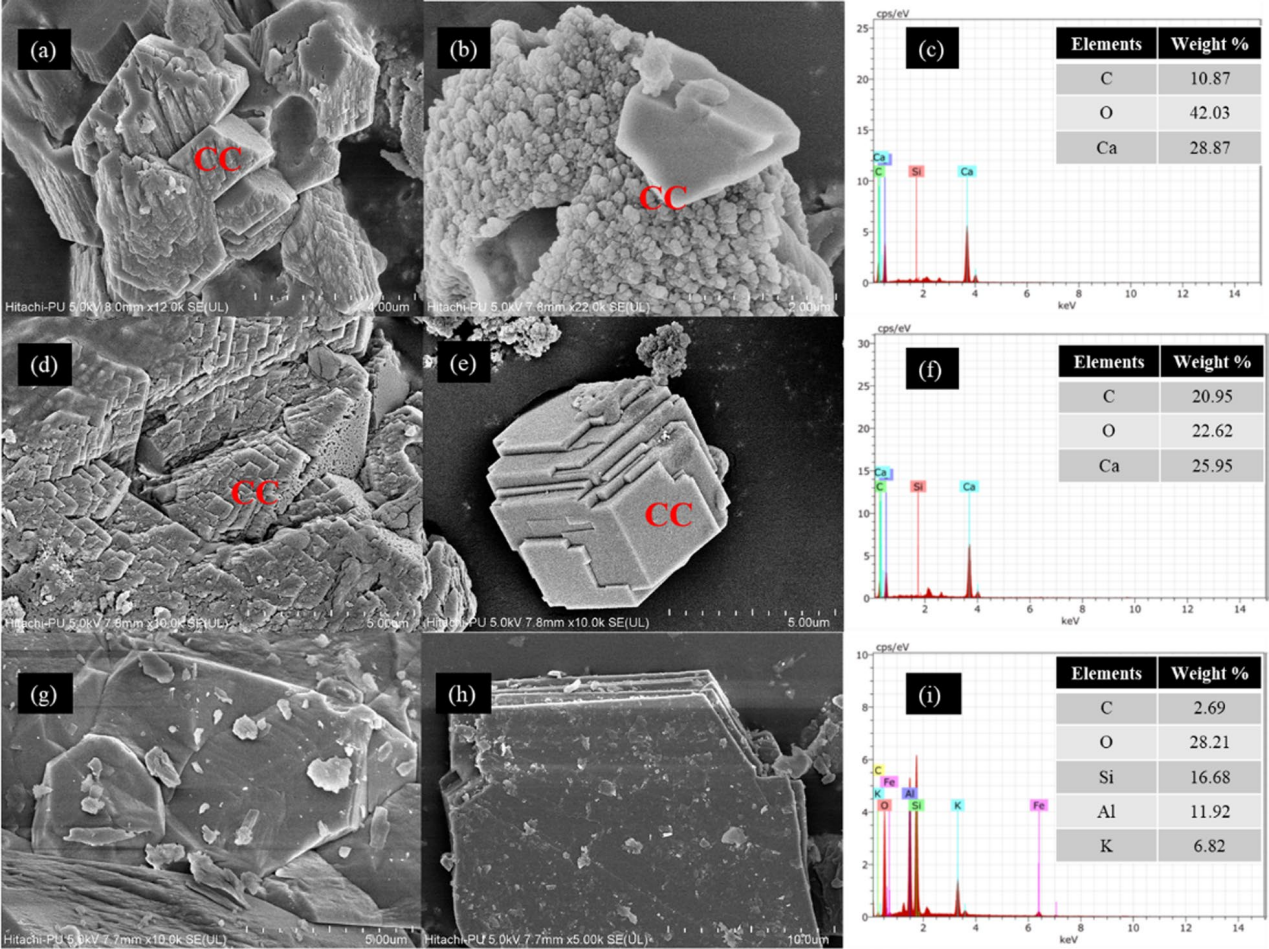
SEM-EDX images represent CaCO_3_ crystals (CC) precipitated on upper layer of sand column treated with live (LT) *Synechocystis pevalekii* cells (**a**, **b**), UV treated (UVT) *S. pevalekii* cells (**d**, **e**), and control (**g**, **h**). EDS analysis (**c**, **f**, **i**) was conducted on the whole area

The upper layer of the sand column was analyzed using SEM-EDS to confirm the calcium carbonate precipitation. Figure 4a and b gives clear confirmation of calcite formation in LT sand column. The precipitation was observed to be dense and bonded to form an aggregation of crystals. Similarly, in Fig. 4d, the staircase of crystals were formed in UVT sand column, which might be due to the accumulation of thick layers of calcite precipitates. The crystals were rhombohedral in shape with a size of 5–7 μm (Fig. 4e). However, no calcite deposition was observed in the control after 14 days of treatments. The precipitated crystal was established to be calcium carbonate polymorph by the elemental analysis of the whole area using energy dispersive spectroscopy (EDS). Figure 4c and f confirm the chemical composition of calcite to be carbon, oxygen, calcium, and silica, which shows the presence of sand particles. These results suggest that *S. pevalekii* cells can precipitate CaCO_3_ crystals within the voids of the sand column.

### Compressive strength

**Fig. 5:**
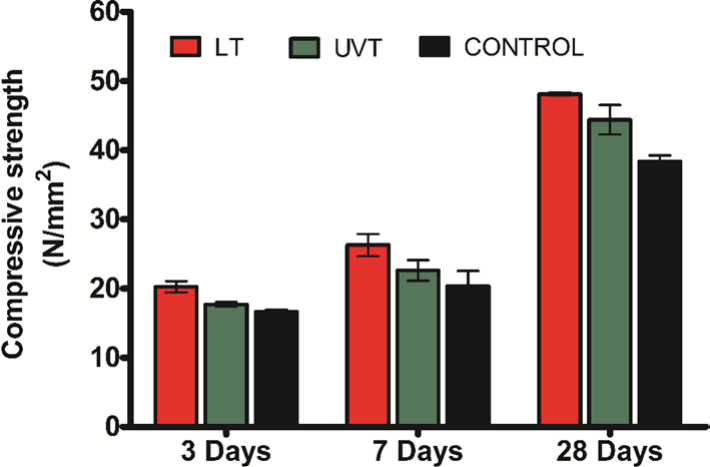
Compressive strength of mortar specimens cured with live (LT) and UV treated (UVT) *S. pevalekii* cells at the age of 3-, 7-, and 28-day

The compressive strength of the mortar specimens was done at different testing ages to study the influence of LT and UVT cells on the cement matrix. The LT and UVT specimens showed improvement in compressive strength, which is attributed to the deposition of CaCO_3_ within the pores of the cement-sand matrix. As shown in Fig. 5, the compressive strength of LT specimens increased from 26.28 MPa at 7-day to 48.11 MPa at 28-day. A similar trend was recorded with UVT specimens, where the compressive strength increased from 22.06 MPa at 7-day to 44.39 MPa at 28-day. The LT specimens observed a 25.54% increase, whereas UVT specimens showed a 15.84% surge in compressive strength after 28 days of curing compared to control specimens.

### Water absorption

**Fig. 6:**
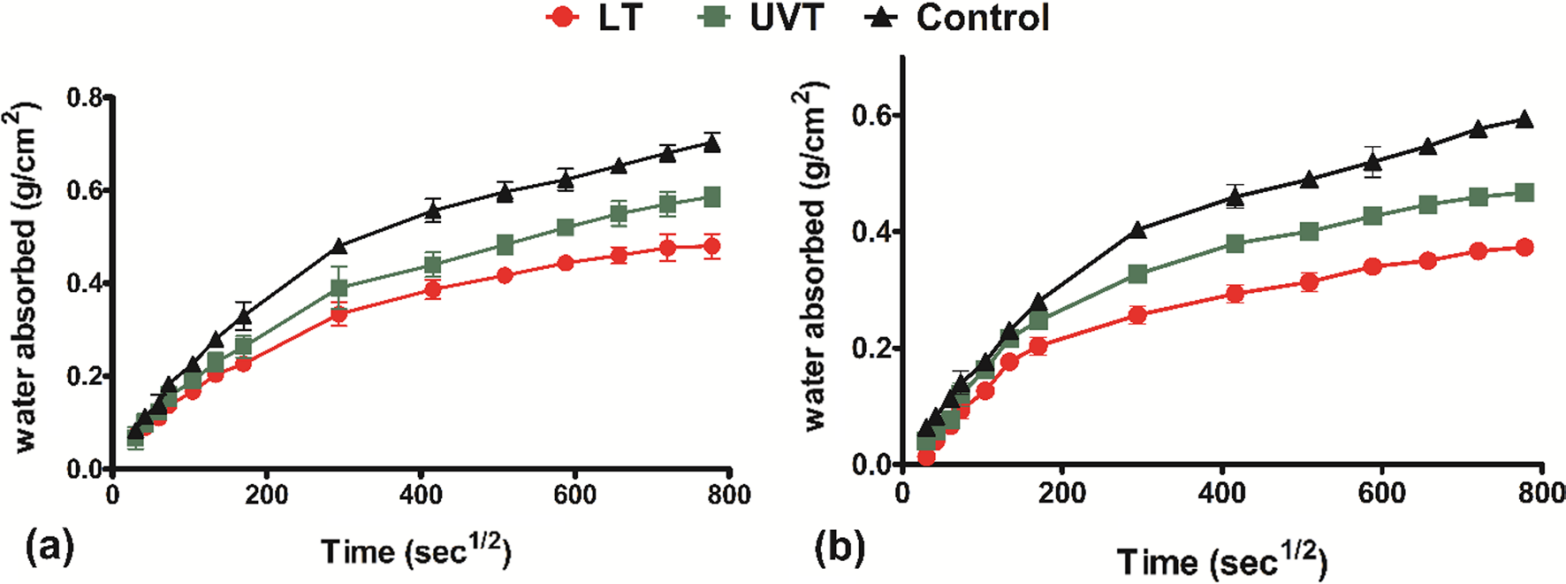
Water absorption of mortar specimens treated with live (LT) and UV treated (UVT) *Synechocystis pevalekii* at **a** 7-day, and **b** 28-day of curing

A water absorption test was performed to determine the water absorption rate by capillary action. The graph representing the cumulative amount of water absorbed per unit cross-sectional area is presented in Fig. 6. The LT and UVT specimens showed lower water absorption, while it was higher in the control specimen. The water absorption was observed to be decreased by 1.5 times in LT specimens with respect to the control after 144 h. Initially, all the mortar specimens exhibited a rapid rise in water absorption until it became constant in the secondary phase of water absorption.

### Thermogravimetric analysis

**Fig. 7:**
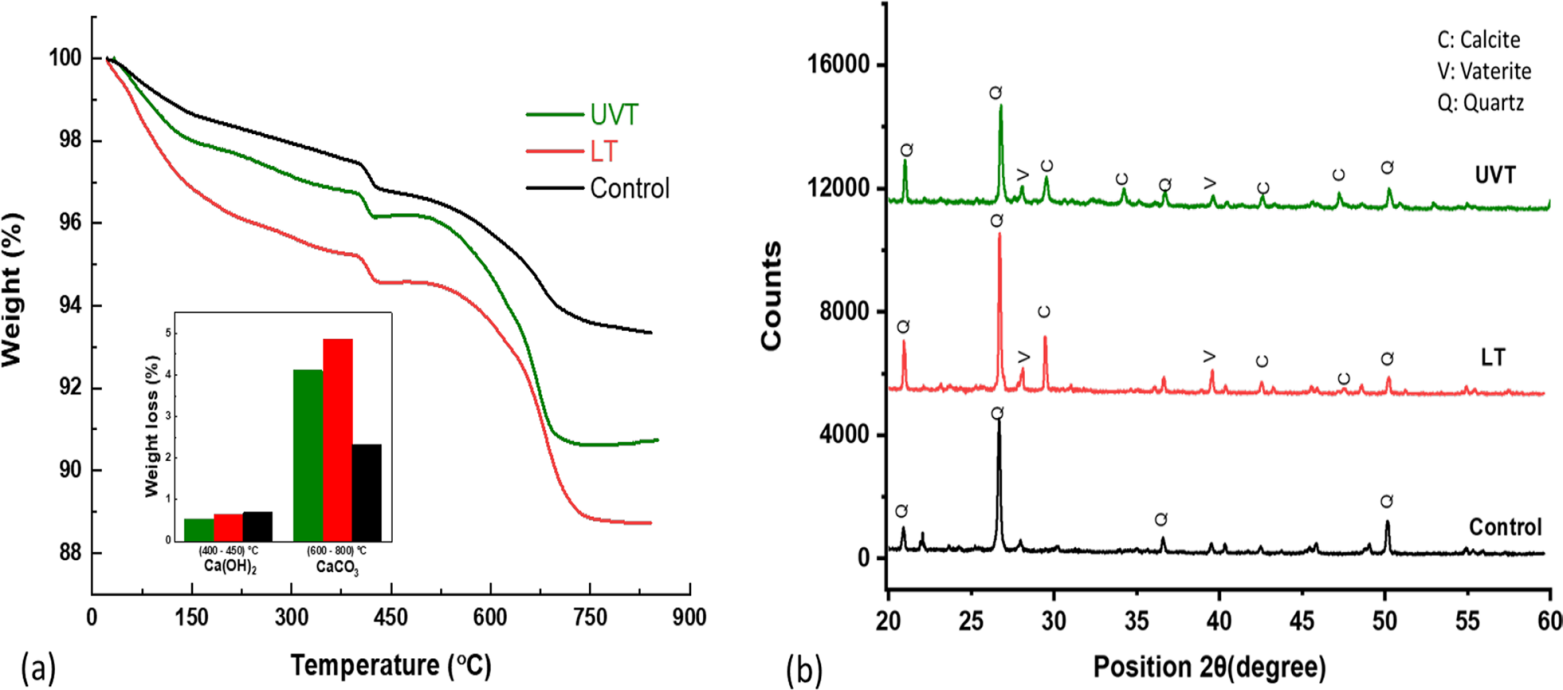
TGA (**a**), and XRD pattern (**b**) of mortar specimens treated with live (LT) and UV treated (UVT) *Synechocystis pevalekii* cells at 28-day of curing

Thermogravimetric analysis (TGA) was conducted for all the specimens at 28-day of curing to confirm the calcium carbonate precipitation. The TGA thermograms for LT, UVT, and control specimens are presented in Fig. 7a. The TGA results showed immense weight loss of 4.86% in LT specimens and 4.09% in the UVT specimens between 600 and 800 °C, corresponding to the carbonate weight loss (Fig. 7a). However, control displayed only 2.32% weight loss percentage between 600 and 800 °C. The TGA’s steeper peaks support the calcium carbonate precipitation by LT and UVT specimens with higher weight loss by LT followed by UVT. Weight loss was comparatively lower in control than LT and UVT specimens. Besides CaCO_3_ decomposition, another fall with 0.63%, 0.52%, and 0.69% weight loss of Ca(OH)_2_ was observed 400–450 °C temperature, showing insignificant mass loss due to dehydration of calcium hydroxide (Fig. 7b).

### Microstructural analysis

The XRD analysis revealed that the control specimen’s prominent crystalline peaks of quartz (SiO_2_) were observed (Fig. 7c). Among all the peaks revealed in the XRD spectra, the calcite polymorph of CaCO_3_ crystals was dominantly present in LT and UVT specimens along with the peak of quartz. XRD spectra of LT specimens were observed at 29.4°, 35.9°, 39.4°, 43.3°, and 48.45° corresponding to the 104, 110, 113, 202, and 116 planes of the calcite phase, whereas in UVT it was observed at 29.4°, 39.4° and 43.3° corresponding to the 10, 110 and 113 planes of the calcite phase (Vu et al. 2020). The diffraction peaks located at (2θ) of 27.1°, 39.6° corresponds to 101, 211 planes of vaterite crystals. The presence of calcite peaks approves the crystal formation by *S. pevalekii* cells in the mortar specimens.

**Fig. 8:**
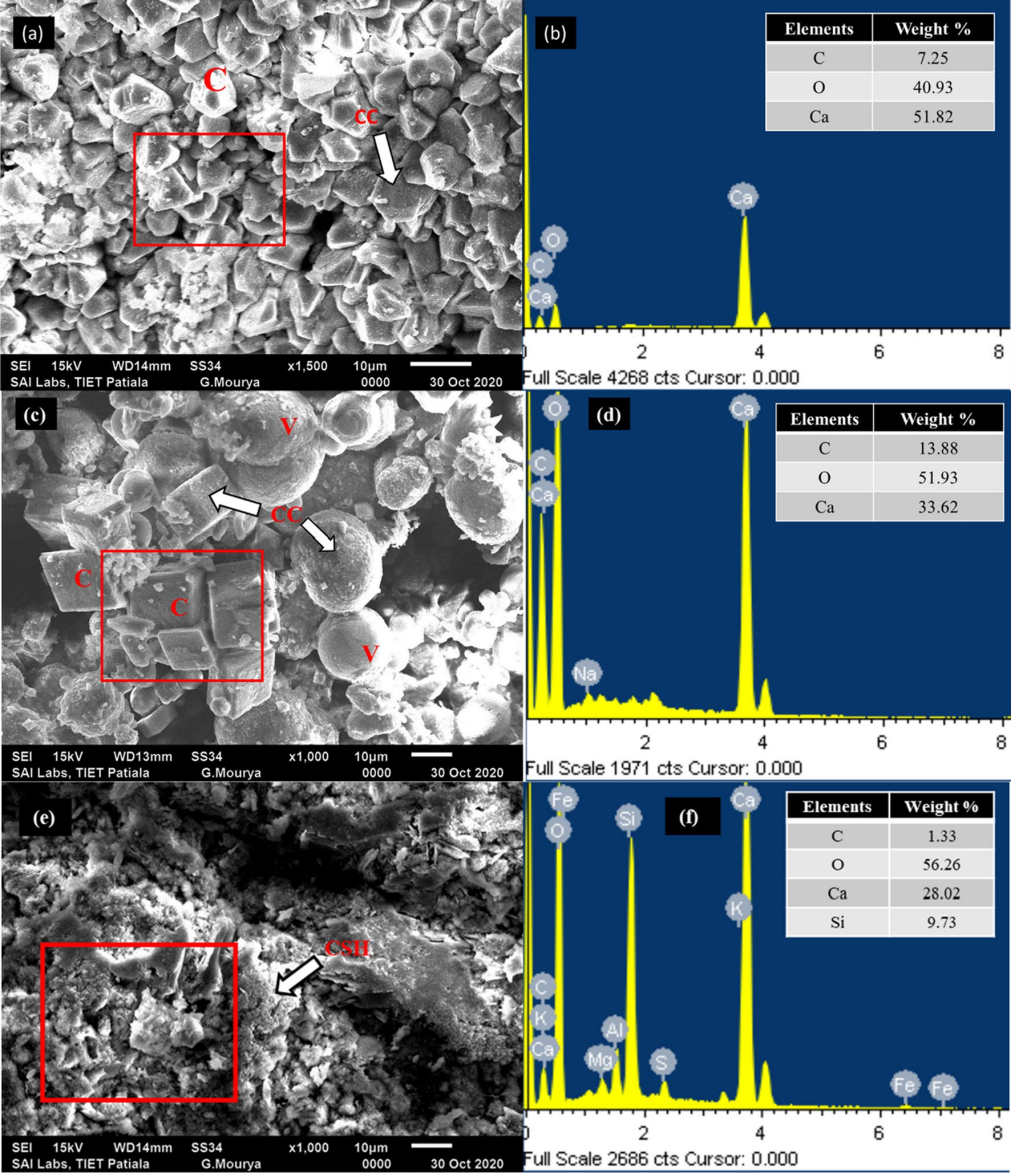
SEM-EDX images represent precipitation of rhombohedral calcite (C) and spherical vaterite (V) CaCO_3_ crystals (CC) in mortar specimens treated with live (LT) *Synechocystis pevalekii* cells (**a**, **b**); UV treated (UVT) *S. pevalekii* cells (**c**, **d**), and control (**e**, **f**). Square ‘□’ sign indicates the spot of EDX analysis

The microstructural analysis of the mortar specimens with LT, UVT, and control specimen are shown in Fig. 8. Figure 8a validates the crystals buildup by UV treated bacterial cells forming a dense layer of calcite over the mortar surface, which was observed to be tightly packed and in a perfect rhombohedral crystal lattice in a size range of 5–10 μm. Likewise, in live treated specimens, the spherical crystal of vaterite ranging in size 17–20 μm were observed along with the cubic crystals of 10–15 μm calcite (Fig. 8b). Both crystals form calcium carbonate, which was confirmed by EDX analysis. The mineral constituents characterized by EDX analysis clearly show a large Ca, O, C peak in LT and UVT specimens compared to the control. As observed in EDX analysis, the amount of carbon, oxygen, and calcium formed in the LT sample was 7.25%, 40.93%, and 51.82%, respectively. Similarly, in the EDX analysis of UVT, the amount of carbon, oxygen, and calcium reported was 13.88%, 51.93%, and 33.62%, respectively, which is a comparatively higher amount than the control.

## Discussion

### Sand consolidation by *S. pevalekii*

Calcium carbonate precipitation by *S. pevalekii* cells plugs the pores between the sand particles. The reduction in the flow rate in LT and UVT treated columns is primarily because of the enclosed biomass and carbonates deposition. The carbonate biominerals reduce the pore size between the sand particles, clogging the flow of calcifying material. The pH of the effluent from the control and the bacterial treated columns showed significant variance so that it can be considered an indicator of the activity of MICCP. Therefore, it can be considered that the most functional element in the calcifying solution is calcium chloride along with sodium bicarbonate which acts as a catalyst for precipitation. Dejong et al. (2010) has reiterated that CaCO_3_ precipitation is an imperative aspect in the distribution of crystals between the sand voids to form bonding between the particles. Sodium bicarbonate used in these columns acts as a catalyst to initiate the formation of calcium carbonate by bacterial cells. Comparatively, minimum calcite content was observed in the bottom part of the columns attributing that growth of *S. pevalekii* cells was promoted in the upper layer of the column due to exposure to the light source and aeration. The results correlated with Dhami et al. (2016), who studied sand consolidation using different grain sizes (0.2, 0.5, and 1.0 mm). They reported an 88% decrease in the flow rate in all bacterial inoculated columns and only a 5% reduction in the control. The effluent pH of bacterial columns was reported to be in the range of 8.5–8.9, whereas the control columns had a pH below 8.1. A similar trend was reported by Achal et al. (2011a), where a decrease in the flow rate and 33% calcite content in the top layer of a sand column. Yang et al. (2020) also reported sand consolidation using urease-producing bacteria. The percentage of calcite precipitated in different layers of the column was in concordance with previous studies (Mahawish et al. 2019; Sharma et al. 2020, 2021).

### Experiment on mortar

The compressive strength of the LT specimen showed improved strength than the UVT with the curing age. This might be due to the slower physiological activity of UV-treated cell culture than live cells. Although UV-treated cells are linked to form stronger adhering to the mortar surface than live cells, live cells actively utilize nutritional media and grow within the medium (Zhu et al. 2018). The photosynthetic reaction by live active cells contributed to the carbonate precipitation by increasing the pH in the microenvironment. In fact, the metabolic active live cells provide more nucleation sites than the dead bacteria since the negative charge on the cell wall is higher and increases their binding capacity (Skevi et al. 2021; Pei et al. 2013) claimed that the live, and dead bacteria have a negligible effect on strength improvement even if nutrient media is provided. On the contrary, Skevi et al. (2021) described 24% and 35% strength enhancement compared to the control by adding live and dead *Bacillus cohii* bacteria. Likewise, Zhu et al. (2017) reported UV-killed specimens showed higher compressive strength than the live cells. However, Heveran et al. (2020) stated negligible compressive strength differences using *Synechococcus* sp. PCC 7002. Srinivas et al. (2021) reported the improvement of the strength of cement mortar treated with microalgae, *Synechococcus elongatus* and *Spirulina platensis*.

The low ‘*k*’ value of the surface-treated specimen indicates that bacterial incorporation can significantly improve the permeation properties. In the beginning, the water absorption test probably results in the rapid filling of large capillary pores near the immersed surface and, finally, a slow rise in the water absorption at the end of testing to more delicate pores far from the submerged surface. Similar results were plotted by Hernández et al. (2016) by incorporating cactus mucilage and marine brown algae extract. Tayebani and Mostofinejad (2019) also reported a reduction in water absorption due to biological treatment compared to water curing.

Along with calcium carbonate, mortar specimens showed weight loss of calcium hydroxide during TGA, formed due to the hydration of cementitious material during the curing process. According to the literature, calcium hydroxide and calcium carbonate dehydration occur at 420–540 °C and 500–800 °C (Stuckrath et al. 2014; Sharma et al. 2017). Thus, this significant drop in calcium carbonate dehydration accounts for higher CaCO_3_ precipitation in bacterial-treated specimens. Therefore, the deposition of calcium carbonate can be considered as an important factor in strength enhancement and permeability reduction. The results were in agreement with the reports of other researchers (Pei et al. 2013; Jang et al. 2020). XRD results are in corroboration with the previous studies showing precipitation of calcium carbonate crystals due to bacterial metabolic activities in the cementitious material (Joshi et al. 2020; Saxena and Tembhurkar 2020).

Our study suggests that incorporating live and UV-treated cyanobacterial cells successfully induced calcium carbonate precipitation in the cementitious environment. It is important to consider that UV treated cells retain the cellular membrane as UV radiations are observed by only purines and pyrimidines of nucleic acid. In heat-killed or autoclaved culture, cell lysis occurs, thus destroying the organic framework of the cell membrane (Malke 1982). Therefore, it is conceivable that besides the slow metabolism of the cell, heterogenous nucleation is promoted by a negative charge on the cell wall, which leads to calcite precipitation in UV-treated cells. At the same time, live cells were able to promote both calcite and vaterite precipitation. The precipitation of calcite and vaterite crystals was observed by Chekroun et al. (2004) in the presence of live and UV-treated *Myxococcus xanthus*. Previous research has well defined the presence of such different morphology and structure of calcium carbonate crystals. The cubic shape of calcite and the spherical shape of vaterite coexist along with the bacterial cell, as reported by Xiao et al. (2010). Different theories have been put forward to illustrate such co-occurrence. Firstly, the vaterite is considered a thermodynamically unstable crystal form, and its nucleation rate is faster; therefore, slow diffusion of Ca^2+^ and CO_3_^2+^ ions can lead to calcite formation (Weiner and Dove 2003). Secondly, the involvement of different organic components of the cell surface provides another nucleation site for biomineralization (Liang et al. 2013; Zhu et al. 2017) reported similar results by treating the specimens with live and UV-killed *Gleocapsa* PCC73106.

In conclusion, calcium carbonate precipitation by *S. pevalekii* cells improved the mortar’s compressive strength and permeation properties in LT and UVT treated specimens compared to control. The compressive strength significantly increased in LT specimens compared to UVT specimens. The microstructural analysis confirmed rhombohedral calcite crystal formation and the bacterial cells in mortar specimens. Thermogravimetric analysis showed a more significant mass loss of carbonate in LT specimens in between 600 and 800 °C than in UVT specimens. Present study results suggest that MICCP by using *S. pevalekii* serves as a low-cost and environment-friendly MICCP technology.

## Data Availability

Data and materials available on request from the corresponding authors.

## References

[CR1] Achal V, Mukherjee A, Reddy MS (2011). Effect of calcifying bacteria on permeation properties of concrete structures. J Ind Microbiol Biotechnol.

[CR2] Achal V, Mukherjee A, Sudhakara Reddy M (2011). Microbial concrete: way to enhance the durability of building structures. J Mater Civ Eng.

[CR3] Bhuvaneshwari T, Shylajanaciyar M, Prakasam PA, Ezhilmaran K, Karthick L, Subramanian G, Uma L, Prabaharan D (2017). Polyphasic analysis for elucidating the taxonomic position of selected unicellular marine* Cyanobacteria* from Indian and Hong Kong coast. Phytotaxa.

[CR4] Chekroun KB, Rodri C, Teresa Gonza M, Maria Arias J, Cultrone G, Rodri M (2004). Precipitation and growth morphology of calcium carbonate induced by *Myxococcus xanthus*: implications for recognition of bacterial carbonates. J Sediment Res.

[CR5] De Muynck W, Verbeken K, De Belie N, Verstraete W (2010). Influence of urea and calcium dosage on the effectiveness of bacterially induced carbonate precipitation on limestone. Ecol Eng.

[CR6] DeJong JT, Mortensen BM, Martinez BC, Nelson DC (2010). Bio-mediated soil improvement. Ecol Eng.

[CR7] Dhami NK, Reddy MS, Mukherjee MS (2013). Biomineralization of calcium carbonates and their engineered applications: a review. Front Microbiol.

[CR8] Dhami NK, Reddy MS, Mukherjee A (2016). Significant indicators for biomineralisation in sand of varying grain sizes. Constr Build Mater.

[CR9] Dittrich M, Sibler S (2010). Calcium carbonate precipitation by cyanobacterial polysaccharides. Geol Soc Spec Publ.

[CR10] Douglas S, Beveridge TJ (2006). Mineral formation by bacteria in natural microbial communities. FEMS Microbiol Ecol.

[CR11] Gnanasekaran DVS (2014). Biocalcification mediated remediation of calcium rich Ossein effluent by filamentous marine cyanobacteria. J Bioremediat Biodegrad.

[CR12] Hamamura N, Olson SH, Ward DM, Inskeep WP (2006). Microbial population dynamics associated with crude-oil biodegradation in diverse soils. Appl Environ Microbiol.

[CR13] Han Z, Yan H, Zhou S, Zhao H, Zhang Y, Zhang N, Yao C, Zhao L, Han C (2013). Precipitation of calcite induced by *Synechocystis* sp. PCC6803. World J Microbiol Biotechnol.

[CR14] Han Z, Zhao Y, Yan H, Zhao H, Han M, Sun B, Meng R, Zhuang D, Li D, Gao W, Du S, Wang X, Fan K, Hu W, Zhang M (2017). The characterization of intracellular and extracellular biomineralization induced by *Synechocystis* sp. PCC6803 cultured under low Mg/Ca ratios conditions. Geomicrobiol J.

[CR15] Hernández EF, De Cano-Barrita PFJ, Torres-Acosta AA (2016). Influence of cactus mucilage and marine brown algae extract on the compressive strength and durability of concrete. Mater Constr.

[CR16] Heveran CM, Williams SL, Qiu J, Artier J, Hubler MH, Cook SM, Cameron JC, Srubar WV (2020). Biomineralization and successive regeneration of engineered living building materials. Matter.

[CR17] IS: 4031 (2005) (Part 6) Methods of physical tests for hydraulic cement part 6 determination of compressive strength of hydraulic cement other than masonry cement (first revision). Bur. Indian Stand. New Delhi-110002

[CR18] IS : 8112 (2013)&nbsp;Indian standard specification for 43 grade ordinary Portland cement, Bur. Indian Stand. New Delhi-110002

[CR19] IS : 383 (2016) Indian standard specification for coarse and fine aggregates from natural sources for concrete, Bur. Indian Stand. New Delhi-110002

[CR20] Jang I, Son D, Kim W, Park W, Yi C (2020). Effects of spray-dried co-cultured bacteria on cement mortar. Constr Build Mater.

[CR21] Joshi S, Goyal S, Sudhakara Reddy M (2020). Influence of biogenic treatment in improving the durability properties of waste amended concrete: a review. Constr Build Mater.

[CR22] Kamennaya NA, Ajo-Franklin CM, Northen T, Jansson C (2012). Cyanobacteria as biocatalysts for carbonate mineralization. Minerals.

[CR23] Kranz SA, Wolf-Gladrow D, Nehrke G, Langer G, Rost B (2010). Calcium carbonate precipitation induced by the growth of the marine cyanobacterium *Trichodesmium*. Limnol Oceanogr.

[CR24] Liang A, Paulo C, Zhu Y, Dittrich M (2013). CaCO_3_ biomineralization on cyanobacterial surfaces: insights from experiments with three *Synechococcus* strains. Colloids Surf B Biointerfaces.

[CR25] Mahawish A, Bouazza A, Gates WP (2019). Unconfined compressive strength and visualization of the microstructure of coarse sand subjected to different biocementation levels. J Geotech Geoenvironmental Eng.

[CR26] Malke H (1982). B. D. Davis, R. Dulbecco, H. N. Eisen and H. S. Ginsberg, microbiology (3rd edition). 1355 S., ca. 1300 Abb., ca. 150 Tab. Hagerstown 1980. Harper & Row Publishers. DFL 65.00. Z Allg Mikrobiol.

[CR27] Paulo C, Kenney JPL, Persson P, Dittrich M (2018). Effects of phosphorus in growth media on biomineralization and cell surface properties of marine cyanobacteria *Synechococcus*. Geoscience.

[CR28] Pei R, Liu J, Wang S, Yang M (2013). Use of bacterial cell walls to improve the mechanical performance of concrete. Cem Concr Compos.

[CR29] Rippka R, Deruelles J, Waterbury JB (1979). Generic assignments, strain histories and properties of pure cultures of cyanobacteria. J Gen Microbiol.

[CR30] Saxena S, Tembhurkar AR (2020). Microbiological induced calcium carbonate process to enhance the properties of cement mortar. Mater Today Proc.

[CR31] Seifan M, Samani AK, Berenjian A (2016). Bioconcrete: next generation of self-healing concrete. Appl Microbiol Biotechnol.

[CR32] Sharma TK, Alazhari M, Heath A, Paine K, Cooper RM (2017). Alkaliphilic *Bacillus* species show potential application in concrete crack repair by virtue of rapid spore production and germination then extracellular calcite formation. J Appl Microbiol.

[CR33] Sharma M, Satyam N, Reddy KR (2020). Strength enhancement and lead immobilization of sand using consortia of bacteria and blue-green algae. J Hazard Toxic Radioact Waste.

[CR34] Sharma M, Satyam N, Reddy KR (2021). Rock-like behavior of biocemented sand treated under non-sterile environment and various treatment conditions. J Rock Mech Geotech Eng.

[CR35] Singh JS, Kumar A, Rai AN, Singh DP (2016). Cyanobacteria: a precious bio-resource in agriculture, ecosystem, and environmental sustainability. Front Microbiol.

[CR36] Skevi L, Reeksting BJ, Hoffmann TD, Gebhard S, Paine K (2021). Incorporation of bacteria in concrete: the case against MICP as a means for strength improvement. Cem Concr Compos.

[CR37] Srinivas MK, Alengaram UJ, Ibrahim S, Phang SM, Vello V, Jun HK, Alnahhal AM (2021). Evaluation of crack healing potential of cement mortar incorporated with blue-green microalgae. J Build Eng.

[CR38] Stocks-Fischer S, Galinat JK, Bang SS (1999). Microbiological precipitation of CaCO_3_. Soil Biol Biochem.

[CR39] Stuckrath C, Serpell R, Valenzuela LM, Lopez M (2014). Quantification of chemical and biological calcium carbonate precipitation: performance of self-healing in reinforced mortar containing chemical admixtures. Cem Concr Compos.

[CR40] Tayebani B, Mostofinejad D (2019). Self-healing bacterial mortar with improved chloride permeability and electrical resistance. Constr Build Mater.

[CR41] Vu HHT, Khan MD, Van Tran T, Quang DV, Dao VD, Lee S, Ahn JW, Jung SH (2020). Use of calcite mud from paper factories in phosphorus treatment. Sustain.

[CR42] Weiner S, Dove PM (2003). An overview of biomineralization processes and the problem of the vital effect. Rev Mineral Geochem.

[CR43] Xiao J, Wang Z, Tang Y, Yang S (2010). Biomimetic mineralization of CaCO_3_ on a phospholipid monolayer: from an amorphous calcium carbonate precursor to calcite via vaterite. Langmuir.

[CR44] Yang Y, Chu J, Cao B, Liu H, Cheng L (2020). Biocementation of soil using non-sterile enriched urease-producing bacteria from activated sludge. J Clean Prod.

[CR45] Zhu T, Lu X, Dittrich M (2017). Calcification on mortar by live and UV-killed biofilm-forming cyanobacterial *Gloeocapsa* PCC73106. Constr Build Mater.

[CR46] Zhu T, Lin Y, Lu X, Dittrich M (2018). Assessment of cyanobacterial species for carbonate precipitation on mortar surface under different conditions. Ecol Eng.

